# The Impact of Obstructive Sleep Apnoea and Nasal Continuous Positive Airway Pressure on Circulating Ischaemia-Modified Albumin Concentrations

**DOI:** 10.1155/2016/8907314

**Published:** 2016-01-20

**Authors:** Firat Uygur, Hakan Tanriverdi, Murat Can, Tacettin Ornek, Fatma Erboy, Bulent Altinsoy, Figen Atalay, Murat Damar, Furuzan Kokturk, Meltem Tor

**Affiliations:** ^1^Faculty of Medicine, Department of Pulmonary Medicine, Bulent Ecevit University, 67600 Zonguldak, Turkey; ^2^Faculty of Medicine, Department of Biochemistry Zonguldak, Bulent Ecevit University, 67600 Zonguldak, Turkey; ^3^Faculty of Medicine, Department of Otolaryngology, Bulent Ecevit University, 67600 Zonguldak, Turkey; ^4^Faculty of Medicine, Department of Biostatistics, Bulent Ecevit University, 67600 Zonguldak, Turkey

## Abstract

The aim of the present study was to evaluate the impact of obstructive sleep apnoea syndrome (OSAS) and the effects of nasal continuous positive airway pressure (CPAP) on circulating ischaemia-modified albumin (IMA) concentrations. The study included 97 newly diagnosed OSAS patients and 30 nonapnoeic controls. Blood samples were obtained in the morning after polysomnography. After 3 months of CPAP treatment, 31 patients with moderate-severe OSAS were reassessed for serum IMA concentrations. Significantly higher serum IMA concentrations were measured in the OSAS group than in the control group [0.518 ± 0.091 absorbance units (ABSU), 0.415 ± 0.068 ABSU, *P* < 0.001]. Serum IMA concentrations correlated significantly with the apnoea-hypopnoea index, mean SaO_2_, desaturation index, and C-reactive protein concentrations. Multiple logistic regression analyses showed that OSAS increased the serum IMA concentration independent of age, sex, body mass index, smoking habit, and cardiovascular disease. After 3 months of treatment with CPAP, OSAS patients had significantly lower serum IMA concentrations (0.555 ± 0.062 ABSU to 0.431 ± 0.063 ABSU, *P* < 0.001). The results showed that OSAS is associated with elevated concentrations of IMA, which can be reversed by effective CPAP treatment.

## 1. Introduction

Obstructive sleep apnoea syndrome (OSAS) is a common disease that develops secondary to the recurrent obstruction of the upper respiratory tract during sleep. OSAS is characterised by episodic hypoxia and arousal [1]. It occurs in 4% of middle-aged males and 2% of middle-aged females [2]. The increase in sympathetic activity caused by repetitive hypoxia and oxidative stress causes cardiovascular and metabolic changes [3, 4]. Thus, OSAS is an important risk factor for cardiovascular diseases (CVDs), such as ischaemic heart disease, arrhythmia, and hypertension [5–7]. In a previous study, we confirmed the strong effects of OSAS on cardiovascular risk factors [8]. As the standard therapy for OSAS, nasal continuous positive airway pressure (CPAP) has been shown to prevent apnoea and related oxygen desaturations; importantly, it also decreases cardiovascular morbidity and mortality [9, 10]. The repetitive episodes of hypoxia and reoxygenation experienced nightly by OSAS patients result in the increased production of reactive oxygen species (ROS) [11]. CPAP therapy was also found to be effective in the prevention of ROS production in patients with OSAS [12, 13].

Ischaemia-modified albumin (IMA) is a sensitive serum marker of myocardial ischaemia [14–16] that has been approved by the U.S. Food and Drug Administration (FDA) for this purpose. Serum IMA concentrations are measured using the albumin cobalt binding method [14]. During ischaemic conditions, the N-terminal region of serum albumin is modified such that its capacity to bind metals (e.g., copper, cobalt, and nickel) is reduced [15]. These changes may be related to ROS production during hypoxia, ischaemia/reperfusion, and acidosis [17]. Because the IMA concentration also increases in various acute ischaemic events, such as skeletal muscle ischaemia, pulmonary embolism, psoriasis, and cerebrovascular disease, it has also been used as a biomarker for the increased risk of CVD [18–24]. Recent research has shown an increase in IMA concentrations under noncardiac oxidative stress conditions [25, 26]. This result is consistent with our own work, in which we showed higher IMA concentrations under noncardiac oxidative stress conditions, including trauma, Alzheimer's disease, and subarachnoid haemorrhage [27–29].

Thus far, only two studies related to OSAS and IMA have been published and only one of them focused on the effect of CPAP therapy [30, 31]. Thus, the aim of this study was to examine IMA in OSAS and to assess the effects of CPAP on the serum concentrations of this marker.

## 2. Material and Methods

### 2.1. Study Population

Patients admitted to the Sleep Centre of the Bulent Ecevit University Hospital, Zonguldak, Turkey, were evaluated prospectively. The study population consisted of 97 consecutive patients with newly diagnosed OSAS and 30 nonapnoeic controls matched for age, sex, body mass index (BMI), and smoking. Patients diagnosed with sleep disorders other than OSAS (e.g., central sleep apnoea syndrome, upper airway resistance, movement disorder, or narcolepsy) and currently treated CPAP patients were excluded from the study. Other exclusion criteria were acute cardiovascular and cerebrovascular ischaemia (e.g., acute coronary syndrome, acute cerebral infarction, and peripheral vascular disease); liver or kidney disease, CVDs, such as coronary artery disease, arrhythmia, or heart failure; thyroid dysfunction; haematological, oncological, or inflammatory diseases; lung diseases characterised by hypoxaemia, such as chronic obstructive pulmonary disease, asthma, and interstitial lung disease; and infection. Patients with drug usage, such as nonsteroidal-anti-inflammatory drugs, steroids, antibiotics, and immunosuppressive medication, as well as alcohol intake, age < 18 years, a history of recent blood transfusion (<2 weeks), or abnormal serum albumin concentrations (<3.5, >5.5 mg/dL), were also excluded.

Data related to the patients' demographic characteristics (age, sex, and BMI), cigarette smoking status, previous history of chronic diseases and drug usage, sleep pattern, and medical history, including cardiovascular and metabolic diseases, were obtained from a standardised questionnaire prior to the sleep study. CVD was defined if the patient had heart failure, coronary artery disease, or arrhythmia. A diagnosis of cardiac disease was confirmed by an expert cardiologist, or if the patient was on one or more of the following: anti-ischaemic agents, beta-blockers, angiotensin-converting enzyme inhibitors, angiotensin-receptor blockers, antiplatelet therapy, or calcium antagonists.

Patients with arterial blood pressure >140/90 mmHg, as measured from the brachial artery after a 5-min resting interval, and those receiving antihypertensive therapy were considered to be hypertensive. Patients with a total cholesterol >200 mg/dL, low density lipoprotein (LDL) cholesterol >130 mg/dL, or triglycerides >150 mg/dL and those using lipid-lowering drugs were considered to be hyperlipidaemic. Excessive daytime sleepiness in patients was assessed by the Epworth Sleepiness Scale (ESS) [32].

This study was approved by the Ethics Committee of Zonguldak Bulent Ecevit University Faculty of Medicine (Zonguldak, Turkey). Written consent was obtained from each participant.

### 2.2. Sleep Study

Each patient underwent full polysomnography (PSG) monitoring at the sleep centre by a technician using two different computer systems (55-channel system, Respironics, USA, and 58-channel system, Compumedics, Australia). At least 6 h of PSG data was recorded. PSG monitoring included an electroencephalogram, electrooculogram, segmental and bilateral leg electromyogram, and an electrocardiogram. Airflow and snoring were measured using an oral thermistor and nasal transducer; thoracic and abdominal wall movements and body position were assessed using inductive plethysmography. Blood oxygen saturation was measured by pulse oximetry. PSG scoring was performed in accordance with the American Academy of Sleep Medicine (AASM) Manual for Scoring Sleep, published by the American Academy of Sleep Medicine in 2012 [33]. After the PSG recording, the sleep stage, changes in heart rate and rhythm, changes in breathing patterns (apnoea, hypopnoea, and arousal), and periodic leg movements during sleep were scored manually. Apnoea was defined as airflow cessation for at least 10 s, and hypopnoea was defined as an airflow reduction of ≥30% for at least 10 s plus an oxygen desaturation of >3% or an arousal. The apnoea-hypopnoea index (AHI) was defined as the total number of hypopnoeas and apnoeas per hour. Based on the AHI, patients were assigned to either the control group or the OSAS group. Patients with an AHI <5 were considered to have simple snoring and assigned to the control group. OSAS was defined as either an AHI ≥ 5 with associated symptoms, such as sleep attacks or excessive daytime sleepiness, unsatisfying sleep, insomnia or fatigue, heavy snoring, and/or breathing pauses as witnessed by the patient's partner, or an AHI ≥ 15 regardless of the associated symptoms. The degree of OSAS was classified according to the AHI as follows: 5 ≤ AHI < 15, mild; 15 ≤ AHI < 30, moderate; and AHI ≥ 30, severe OSAS.

### 2.3. CPAP Titration and Therapy

CPAP was titrated manually by a technician using two devices (Respironics, Murrysville, PA, USA, and Weinmann, Hamburg, Germany), with the patient under full PSG monitoring. CPAP titration was started with the pressure set at 4 cm H_2_O under full-night PSG and was increased incrementally until apnoea-hypopnoea events disappeared. The lowest pressure that eliminated these events was considered the optimal pressure. A titration study was performed for at least 6 h. All participants had sufficient sleep efficiency (>70%). After 3 months of treatment with nasal CPAP, these patients underwent clinical reassessment and a biochemical analysis. Treatment compliance was measured using the built-in data stores of the CPAP device. At least 5 h/night for at least 70% of the nights/week was defined as acceptable CPAP therapy.

### 2.4. Ischaemia-Modified Albumin Assay

Blood samples were drawn from the patients between 8:00 AM and 9:00 AM, after they had undergone a baseline PSG. After 3 months of treatment, a further serum sample was obtained. Subjects were required to fast before blood samples were collected. The blood samples were centrifuged within 30 min at 4°C at 3000 g for 10 min. Serum samples were stored at −80°C until used in the assay. Serum IMA concentrations were measured with a colourimetric method in which reduced cobalt binding to albumin is measured, as described by Bar-Or et al. [14]. The results were expressed in absorbance units (ABSU). The assay has an intra-assay coefficient of variation (%CV) of <3.5% and an interassay %CV of <6.1%. The serum C-reactive protein (CRP) concentration was measured as highly sensitive CRP (CRP) in an immunoturbidimetric assay using the CRP reagent and a Beckman Coulter AU2700 analyser (Beckman Coulter, Inc., Fullerton, CA USA).

### 2.5. Statistical Analysis

Statistical analyses were performed with SPSS 19.0 software (SPSS Inc., Chicago, IL, USA). The distribution of the data was determined using the Shapiro-Wilk test. Data are expressed as the mean ± standard deviation, and categorical variables are expressed as the frequency and percent. Continuous variables were compared to the independent sample *t*-test or the Mann-Whitney *U* test for two groups. Categorical variables were compared using Pearson's *χ* ^2^ test or Fisher's exact test. An ANOVA or the Kruskal-Wallis test was used to determine the differences between three or more groups. The Tukey test was used as a post hoc test, if the ANOVA results were statistically significant. Dunn's test was used as the post hoc test after the Kruskal-Wallis test. A receiver operating characteristic (ROC) analysis was performed to determine the best cut-off value to predict the outcome. Multiple logistic regression was used to identify independent predictors of OSAS. For all tests, a *P* value <0.05 was considered to indicate statistical significance.

## 3. Results

The 127 participants enrolled in this study had a mean age of 51.7 ± 10.7 years and 90 (71%) were male. Their baseline clinical, laboratory, and PSG findings are listed in Table 1. The study population was divided into four groups based on the AHI scores: control (group 1, *n* = 30), mild OSAS (group 2, *n* = 32), moderate OSAS (group 3, *n* = 31), and severe OSAS (group 4, *n* = 34). There were no significant differences between the patients with OSAS and the controls in terms of age, sex, BMI, smoking status, hypertension, or diabetes mellitus status.

Serum IMA concentrations were higher in the combined OSAS groups than in the control group (0.518 ± 0.091 ABSU versus 0.415 ± 0.068 ABSU, *P* < 0.001). In addition, serum IMA concentrations differed significantly among patients with mild, moderate, and severe OSAS (0.457 ± 0.077 ABSU, 0.516 ± 0.089 ABSU, and 0.577 ± 0.063 ABSU, resp., *P* < 0.05) (Table 1). Plasma CRP concentrations were also significantly higher in all three OSAS groups combined than in the control group (3.22 ± 1.2 mg/L and 1.56 ± 1.2 mg/L, *P* < 0.001).

**Figure 1:**
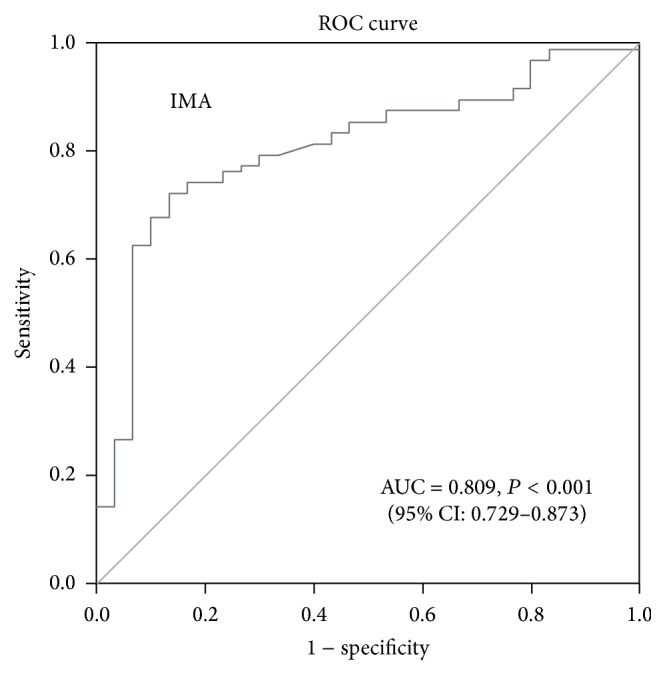
Receiver operating characteristics (ROC) curve analysis for cut-off value of 0.467 ABSU for IMA. IMA: ischaemia-modified albumin; AUC: area under the curve; CI: confidence interval.

According to a ROC analysis of serum IMA, the ROC rederived cut-off for IMA was 0.467 ABSU [AUC: 0.809; *P* < 0.0001, 95% confidence interval (CI): 0.729–0.873, 72.2% sensitivity, 86.6% specificity] (Figure 1). We therefore divided the entire group of patients into two groups in terms of their serum IMA concentration. Thus, patients with an IMA concentration >0.467 ABSU were assumed to be IMA-positive and all others were IMA-negative. The results of multiple logistic regression analyses including age, sex, BMI, smoking status, hypertension, and IMA group (IMA-negative or IMA-positive according to the cut-off value of 0.467 ABSU) are summarised in Table 2. A positive IMA value [odds ratio (OR): 10.838; 95% CI: 3.663–32.068, *P* < 0.001] was an independent predictor of OSAS.

A Spearman correlation analysis revealed a significant correlation between serum IMA concentrations and the AHI (*r* = 0.471, *P* < 0.001), mean SaO_2_ (*r* = −0.555, *P* < 0.001), desaturation index (*r* = 0.493, *P* < 0.001), ESS score (*r* = 0.500, *P* < 0.001), and CRP concentration (*r* = 0.518, *P* < 0.001).

After 3 months of CPAP treatment, 31 patients with moderate-severe OSAS were reassessed for serum IMA concentrations (Table 3). Average CPAP use in this group was 6.45 ± 2.73 h/night for at least 5 nights per week. The CPAP titration pressure ranged from 5 to 12 cm H_2_O (mean = 7.6 ± 1.82 cm H_2_O). The results showed a significant decrease in serum IMA concentrations (from 0.555 ± 0.062 ABSU to 0.431 ± 0.063 ABSU, *P* < 0.001) and in serum CRP concentrations after 3 months of CPAP treatment (Table 3).

## 4. Discussion

Oxidative stress and inflammation are two important mechanisms underlying the pathophysiology of OSAS. However, the increased oxidative stress induced by hypoxia-related free radicals and intermittent hypoxia [34] and by increased ROS production is reversed by CPAP therapy [13]. Similarly, various studies have shown that both the total antioxidant capacity and the serum concentrations of vitamins A and E are diminished in patients with OSAS, but they improve following CPAP treatment [35].

ROS can transiently alter the N-terminal region of albumin and increase the concentration of IMA. Thus, serum IMA provides an ideal marker to assess ischaemia and therefore the risk of CVD as well [18–20]. Moreover, several studies have shown an increase in IMA during noncardiac oxidative stress conditions, such as chronic kidney disease, systemic sclerosis, and Alzheimer's disease [25, 36–39].

Our patients with OSAS had higher serum IMA concentrations than the control group. However, in the former, 3 months of CPAP therapy resulted in a significant decrease in serum IMA concentrations. Moreover, the circulating IMA concentration correlated positively with the serum CRP concentration. In contrast, Ozben et al. measured serum IMA concentrations in OSAS patients [30] and did not find a significant relation between IMA and OSAS. However, Yang et al. [31] reported higher IMA values in OSAS patients than in control subjects. Our study differed from these studies [30, 31] with respect to the study population, methodology (duration of CPAP therapy), and exclusion criteria. The patients of Yang et al. had mild OSAS; OSAS was classified according to AHI, and the presence of ischaemic diseases was not excluded. Among our patients, serum IMA concentrations were higher in those with OSAS than in the controls. Moreover, IMA correlated positively with the AHI, desaturation index, ESS, and CRP and negatively with the mean SaO_2_. However, Yang et al. found a positive correlation only between IMA and AHI, but not between IMA and other variables that characterise OSAS patients [31].

CPAP therapy prevents the narrowing and collapse of the soft tissue of the upper airway during sleep and thus improves OSAS-associated hypoxia/reoxygenation, sympathetic activity, inflammation, and the resulting consequences [40, 41]. Serum IMA concentrations decreased in the 9 OSAS patients of Yang et al. after 4 weeks of CPAP therapy [31] and in our OSAS patients after 3 months of CPAP therapy. Taken together, these results show that a change in IMA concentrations is a valuable predictor in OSA treatment monitoring.

As an indicator of a systemic inflammatory response, CRP is a significant marker of inflammation. Elevated CRP concentrations were measured in our patients with OSAS, but the precise mechanisms underlying the relationship between CRP and OSAS are not fully understood [8]. Furthermore, whether or not CPAP therapy has an effect on CRP concentrations is controversial, as some studies have shown a favourable outcome [42, 43], but others found no significant change [44, 45]. In our study, serum CRP concentrations decreased significantly following CPAP therapy for OSAS.

OSAS increases the serum concentrations of IMA independent of age, sex, BMI, smoking habit, and cardiovascular disease. The serum IMA cut-off value for OSAS in our study was 0.467 ABSU; a value above this concentration was an independent predictor of OSAS.

Our study had several limitations. In addition to the small sample size, we included patients without coronary artery disease, even though it is not uncommon in OSAS. Individuals in the control and OSAS groups were examined for the presence of cardiac disease based on their medical history, blood pressure values, a physical examination, and electrocardiography, but they did not undergo specific tests of cardiac function. Consequently, in both the control and the OSAS group, asymptomatic coronary artery disease may have gone undetected. Finally, a major limitation of the study was the limited number of patients who underwent CPAP therapy and were then followed longitudinally.

## 5. Conclusion

The present study showed that serum IMA concentrations are higher in OSAS patients than in healthy subjects but decrease significantly in the former with effective CPAP therapy. We therefore recommend the use of increased serum IMA concentrations as an independent predictive marker of the presence and severity of OSAS as well as its reversal in patients who respond to CPAP.

## Figures and Tables

**Table 1:** Demographic, clinical characteristics and laboratory values of the study group.

	Group 1 Control group (*n* = 30)	Group 2 Mild OSAS (*n* = 32)	Group 3 Moderate OSAS (*n* = 31)	Group 4 Severe OSAS (*n* = 34)	*P*
Age (year)^a^	49.2 ± 13.1	53 ± 7.8	51.7 ± 11.5	52.6 ± 9.8	0.570
Male/female gender	21/9	23/9	19/12	27/7	0.456
BMI (kg/m^2^)^a^	28.9 ± 4.4	29.4 ± 4.5	30.1 ± 3.5	31.4 ± 4.7	0.091
Smoker/nonsmoker	14/16	12/20	14/17	14/20	0.884
Comorbidities					
Hypertension	7	8	11	17	0.095
Diabetes mellitus	3	3	5	7	0.518
Hyperlipidemia	6	9	8	10	0.839
Polysomnographic study results					
Total sleep time (TST), h	6.7 ± 1.3	6.2 ± 1.1	6.4 ± 1.2	6.1 ± 0.9	0.620
Sleep efficiency (%)	84.7 ± 10.3	84.7 ± 9.5	83.8 ± 10.6	84 ± 9.8	0.974
Stages 3, % of TST	21.4 ± 6.4^b^	15.4 ± 10	12.9 ± 7.9	9.2 ± 4.8	<0.001
REM, % of TST	23.7 ± 4.1^c^	18.3 ± 5.9^d^	15.8 ± 5.2	11.8 ± 5.6	<0.001
AHI, h	1.8 ± 1.3^b^	8.6 ± 2.1^c^	18.8 ± 5^d^	50 ± 18.8	<0.001
Mean oxygen saturation, %	93.2 ± 3^b^	88.8 ± 3.5^c^	83.5 ± 4.7^d^	73 ± 9.2	<0.001
Desaturation index	4.16 ± 4.2^b^	14.3 ± 8^c^	26.4 ± 26.6^d^	59.7 ± 25.7	<0.001
ESS	4.7 ± 2.3^b^	8.8 ± 3^c^	11.2 ± 3.5^d^	15.8 ± 3.8	<0.001
IMA (ABSU)	0.415 ± 0.068^b^	0.457 ± 0.077^c^	0.516 ± 0.089^d^	0.577 ± 0.063	<0.001
CRP (mg/L)	1.56 ± 1.2^b^	2 ± 1^c^	3.1 ± 1.4	4.4 ± 1.8	<0.001

OSAS: obstructive sleep apnea syndrome, BMI: body mass index, REM: rapid eye movement, AHI: apnea-hypopnea index, ESS: Epworth Sleepiness Scale, IMA: ischaemia-modified albumin, and CRP: C-reactive protein.

^a^Data are presented as means ± 1 standard deviation.

^b^Statistically significantly different from groups 2, 3, and 4.

^c^Statistically significantly different from groups 3 and 4.

^d^Statistically significantly different from group 4.

**Table 2:** Independent predictors of obstructive sleep apnoea syndrome in multivariate logistic regression analysis.

	OR	95% CI	*P* value
Age	1.0	0.96–1.06	0.65
Sex	0.9	0.52–1.73	0.79
Smoking	0.8	0.31–2.12	0.68
BMI > 25 (kg/m^2^)	1.0	0.88–1.15	0.85
Hypertension	1.7	0.45–6.86	0.41
IMA positive group	10.8	3.66–32.07	<0.001

OR: odds ratio; CI: confidence interval; BMI: body mass index; IMA: ischaemia-modified albumin; patients with IMAs >0.467 were assumed to be IMA-positive and all others were IMA-negative.

**Table 3:** Alterations in serum IMA and CRP concentrations and demographic information after 3 months of CPAP therapy.

	Pre-CPAP (*n* = 31)	Post-CPAP	*P*
Age (year)	56.3 ± 8		
Male/female gender	22/9		
BMI (kg/m^2^)	31.5 ± 4.2	31.6 ± 4.2	0.459
IMA (ABSU)	0.555 ± 0.062	0.431 ± 0.063	<0.001
CRP (mg/L)	3.97 ± 1.4	1.72 ± 1	<0.001

Data are expressed as the mean ± SD.

IMA: ischaemia-modified albumin, CPAP: continuous positive airway pressure, BMI: body mass index, and CRP: C-reactive protein.
